# Loss- and Gain-of-Function Approaches Indicate a Dual Role Exerted by Regulatory T Cells in Pulmonary Paracoccidioidomycosis

**DOI:** 10.1371/journal.pntd.0004189

**Published:** 2015-10-29

**Authors:** Silvia B. Bazan, Tania A. Costa, Eliseu Frank de Araújo, Claudia Feriotti, Flávio V. Loures, Fernando D. Pretel, Vera L. G. Calich

**Affiliations:** 1 Departamento de Imunologia, Instituto de Ciências Biomédicas, Universidade de São Paulo, São Paulo, Brazil; 2 Centro de Facilidades de Apoio à Pesquisa (CEFAP), Instituto de Ciências Biomédicas, Universidade de São Paulo, São Paulo, Brazil; University of California, San Diego School of Medicine, UNITED STATES

## Abstract

Paracoccidioidomycosis (PCM), is a pulmonary fungal disease whose severity depends on the adequate development of T cell immunity. Although regulatory T (Treg) cells were shown to control immunity against PCM, deleterious or protective effects were described in different experimental settings. To clarify the function of Treg cells in pulmonary PCM, loss-and gain-of-function approaches were performed with Foxp3^GFP^ knock-in mice and immunodeficient Rag1^-/-^ mice, respectively, which were intratracheally infected with 10^6^ yeast cells. The activity of Foxp3-expressing Treg cells in pulmonary PCM was determined in Foxp3^GFP^ transgenic mice. First, it was verified that natural Treg cells migrate to the lungs of infected mice, where they become activated. Depletion of Treg cells led to reduced fungal load, diminished pathogen dissemination and increased Th1/Th2/Th17 immunity. Further, adoptive transfer of diverse T cell subsets to Rag1^-/-^ mice subsequently infected by the pulmonary route demonstrated that isolated CD4^+^Foxp3^+^ Treg cells were able to confer some degree of immunoprotection and that CD4^+^Foxp3^-^ T cells alone reduced fungal growth and enhanced T cell immunity, but induced vigorous inflammatory reactions in the lungs. Nevertheless, transfer of Treg cells combined with CD4^+^Foxp3^-^ T cells generated more efficient and balanced immune Th1/Th2/Th17 responses able to limit pathogen growth and excessive tissue inflammation, leading to regressive disease and increased survival rates. Altogether, these loss- and gain-of-function approaches allow us to clearly demonstrate the dual role of Treg cells in pulmonary PCM, their deleterious effects by impairing T cell immunity and pathogen eradication, and their protective role by suppressing exacerbated tissue inflammation.

## Author Summary

Paracoccidioidomycosis (PCM), the most relevant deep mycosis in Latin America, is caused by the fungus *Paracoccidioides brasiliensis*. The involvement of regulatory T (Treg) cells in the immunity against PCM has been previously demonstrated, however its underlying mechanisms are still to be elucidated. Using Foxp3^GFP^ transgenic mice, we found that natural Tregs migrate to the lungs of infected mice, where they become activated. Depletion of Treg cells led to reduced fungal load, diminished pathogen dissemination and increased Th1/Th2/Th17 immunity. Further, after performing adoptive transfer experiments using immunodeficient Rag1^-/-^ mice, we demonstrated that isolated CD4^+^Foxp3^+^ Treg cells were able to confer some degree of immunoprotection and that CD4^+^Foxp3^-^ T cells alone contributed to fungal elimination and enhanced T cell immunity, but induced vigorous inflammatory reactions in the lungs. Nevertheless, transfer of Tregs combined with CD4^+^Foxp3^-^ T cells generated more efficient and balanced immune responses able to limit pathogen growth and excessive tissue inflammation, leading to regressive disease and higher survival rates. Altogether, these loss- and gain-of-function approaches allow us to propose a dual role for Treg cells in pulmonary PCM, their deleterious effects by impairing T cell immunity and pathogen eradication, and their protective role by suppressing exacerbated tissue inflammation.

## Introduction

Regulatory T cells (Tregs) were initially considered a Foxp3-expressing CD4^+^CD25^+^ T cell subset involved in maintenance of immune tolerance by suppressing T cell responses against self-antigens. Nevertheless, recent studies have also clearly revealed their crucial role in modulating immunity during infection by a number of pathogens [reviewed by 1,2]. Although many studies associate the presence of Tregs with deleterious effects on effector T cells and disease outcome in diverse models of infection, suppression triggered by Tregs has been demonstrated to be protective in certain infectious diseases in which tissue damage derived from excessive inflammation surpasses the injury induced by the pathogen [3,4].

Although the involvement of Tregs in a series of viral, bacterial and parasitic infections has been widely studied, their participation in fungal pathologies has also been recently documented, such as in infections caused by *Pneumocystis carinii*, *Candida albicans* and *Histoplasma capsulatum* [4–6]. The function of Tregs in immunity against *P*. *brasiliensis*, which causes a chronic granulomatous systemic mycosis in Latin America, has only been investigated in recent years. In human paracoccidioidomycosis (PCM), a study has shown an augmented number of CD4^+^CD25^+^Foxp3^+^ T cells in the lesions and in the peripheral blood of infected patients, and these cells exhibited strong suppressive activity [7]. In the murine model of PCM, some studies have demonstrated the deleterious effects of Tregs on PCM, while others have suggested protective role of Treg cells. Moreira (2008) et al. demonstrated that CCR5-deficient mice showed impaired Treg cell migration to pulmonary lesions, more compact granulomas and controlled fungal dissemination [8]. Our group has shown that Treg depletion led to a less severe infection in both *P*. *brasiliensis*-resistant and -susceptible mouse strains, since antibody-mediated Treg ablation rescued susceptible animals from progressive disease and precocious mortality and significantly reduced organ pathology in both mouse strains [9]. In a more recent study, we have shown increased expansion of Treg cells in dectin^-/-^ mice infected with *P*. *brasiliensis*, which paralleled a diminished activation and migration of CD4^+^ and CD8^+^ T cells to the site of infection, resulting in a more severe disease outcome [10]. In other experimental settings, unrestrained inflammatory responses were observed in mice with impaired Treg cell expansion, leading to more severe tissue pathology [11,12].

In order to further investigate the mechanisms underlying Treg cell activity, we used Foxp3^GFP^ transgenic mice to track the development and function of Foxp3-expressing Tregs both in acute and chronic stages of pulmonary PCM. We further explored the role of Treg cells in PCM using complementary loss- and gain-of-function approaches, either by depleting Tregs with monoclonal antibodies or by reconstituting Rag1^-/-^ mice with this cell subset. Herein, we show that Treg cells residing in pulmonary lesions display a natural Treg (nTreg) phenotype, become activated in the site of infection and secrete the suppressive cytokine IL-10. Further, Treg cell depletion with anti-CD25 led to increased influx of activated CD4^+^ and CD8^+^ T cells at the site of infection, enhanced Th1, Th2 and Th17 responses and reduced tissue pathology. Conversely, reconstitution of Rag1^-/-^ mice with naïve CD4^+^Foxp3^-^ T cells led to efficient fungal clearance but exacerbated inflammation in the lungs, resulting in higher mortality rates in comparison with mice given CD4^+^Foxp3^-^ T cells in combination with Tregs, which exhibited regressive pathology associated with mixed Th1/Th2/Th17 immune responses, culminating in enhanced survival times. Thus, our findings provide for the first time direct evidence for a dual role of Tregs in host defense against PCM.

## Methods

### Ethics statement

Animal experiments were performed in strict accordance with the Brazilian Federal Law 11,794 establishing procedures for the scientific use of animals, and the State Law establishing the Animal Protection Code of the State of São Paulo. All efforts were made to minimize suffering, and all animal procedures were approved by the Ethics Committee on Animal Experiments of the Institute of Biomedical Sciences of University of São Paulo (Proc.76/04/CEEA).

### Mouse strains

C57BL/6 Foxp3^GFP^ and C57BL/6 Rag1^-/-^ mice were bred and maintained at the University of São Paulo animal facilities under specific-pathogen-free (SPF) conditions in microisolator cages. Male mice were used at 8 to 12 weeks of age and received sterilized rodent chow and water *ad libitum*.

### Fungus and infection

The highly virulent *P*. *brasiliensis* 18 isolate (Pb18) was used throughout this study. To ensure the maintenance of its virulence, the isolate was used after three serial animal passages [13]. Yeast cells were maintained by weekly subcultivation in semisolid Fava Netto culture medium [14] at 36°C and used on days 5–7 of culture. For infection studies, fungal particles were washed in PBS, counted and adjusted to 20 × 10^6^ cells ml^-1^. Individual cell counts were used after extensive elimination of clumped cells by spontaneous sedimentation, followed by buds disruption after repeated passages of the fungal suspension by a tuberculin syringe connected to a hypodermic needle. The viability of fungal suspensions, determined by Janus Green B vital dye (Merck), was always higher than 85%. Mice were anesthetized and submitted to intra-tracheal (i.t.) *P*. *brasiliensis* infection as previously described [15]. Briefly, after intraperitoneal injection of ketamine and xylazine, animals were infected with 1×10^6^ Pb18 yeast cells, contained in 50 mL of PBS, by surgical i.t. inoculation, which allowed dispensing of the fungal cells directly into the lungs. The skin was then sutured, and mice were placed under a heat lamp until they recovered from anesthesia.

### Treg cell depletion

I*n vivo* depletion of Treg cells with anti-CD25 antibodies was performed as previously described [9]. We verified that this schedule was very effective in the depletion of Treg cells without causing significant alterations in other T cell subsets. Briefly, C57BL/6 Foxp3^GFP^ mice were given i.p. injections of 500 μg of anti-CD25 (clone PC61) or control rat IgG (BioXcell, USA) diluted in sterile PBS. Antibodies were administered on days -3 and +3 relative to infection with *P*. *brasiliensis* yeasts.

### Cell sorting and adoptive cell transfer

Leukocytes were obtained from spleens of Foxp3^GFP^ mice. After lysis of erythrocytes, splenocytes were enriched for CD4^+^ T lymphocytes using magnetic beads (Miltenyi Biotec) according to the manufacturer’s instructions. Following separation, CD4^+^ T cells were stained with anti-CD4 APC (BD Biosciences) and sorted into CD4^+^Foxp3^GFP+^ and CD4^+^Foxp3^GFP-^ populations using a FACSAria cell sorter (BD Biosciences). The sorted cell populations were routinely > 98% pure. Rag1-deficient mice were injected intravenously with 2 × 10^6^ CD4^+^Foxp3^GFP-^, 2 × 10^5^ CD4^+^Foxp3^GFP+^, or a combination of both cell subsets, in 100 μl sterile PBS 24 h prior to infection with Pb18.

### Colony forming units (CFU) assays

To assess the viable number of CFU in target organs, lungs, livers and spleens from Foxp3^GFP^ and Rag1^-/-^ mice were aseptically removed, weighted and homogenized in 5 ml PBS using tissue grinders as previously described [16]. Next, 100 μL aliquots of 50- and 100-fold dilutions from organs were plated onto petri dishes containing brain heart infusion agar (Difco) supplemented with 5% *P*. *brasiliensis* 192 culture filtrate and 4% (v/v) horse serum (Instituto Butantan, São Paulo, Brazil), and incubated at 36°C. Colonies were counted until no increase in counts was observed and CFU per gram of tissue were determined.

### Mortality rates

Mortality studies were performed with Rag1^-/-^ mice receiving the different cell subsets or PBS and inoculated i.t. with 1×10^6^ yeast cells. Deaths were registered daily and the mean survival time after infection was calculated.

### Histopathological analysis

Lungs, liver and spleen from Foxp3^GFP^ and Rag1^-/-^ mice were collected, fixed in 10% formalin and embedded in paraffin. Sections of 5 μm were stained with hematoxilin-eosin (H&E) for analysis of the lesions and Grocott for fungal evaluation. Pathology was analyzed based on the size, morphology and cell composition of granulomatous lesions, presence of fungi and intensity of the inflammatory infiltrates. Morphometrical analysis was performed using a Nikon DXM 1200c digital camera and Nikon NIS Elements AR 2.30 software. Results are expressed as the mean ± standard deviation (SD) for the total area of lesions.

### Flow cytometry analysis

For cell-surface staining, leukocytes were washed and resuspended at 1 × 10^6^ cells/mL in staining buffer (PBS, 2% fetal calf serum and 0.1% NaN_3_). Fc receptors were blocked by the addition of unlabeled anti-CD16/32 (Fc block; BD Pharmingen). Leukocytes were then stained in the dark for 20 min at 4°C with the optimal dilution of each monoclonal antibody: Brilliant Violet 510 (BV)-labeled anti-CD45; Pacific Blue (PB)-labeled anti-CD4, anti-IL-10; phycoerythrin (PE)-labeled anti-CD44, anti-CTLA-4, anti-CD73, anti-CCR5, anti-IL-17, anti-Ly6G; PE-Cy5-labeled anti-CD69; PECy7-labeled anti-CD25, anti-PD-1, anti-CD39, anti-TNF-α, anti-IL-4; peridinin chlorophyll protein (PerCP)-labeled anti-CD25, anti-ICOS, anti-IFN-γ; allophycocyanin (APC)-labeled anti-CD8, anti-GITR, anti-Neuropilin-1, anti-Helios, anti-F4/80; APC-Cy7-labeled anti-CD4, anti-CD62L (from BD Biosciences or BioLegend). Cells were washed twice with staining buffer, fixed with 1% paraformaldehyde (Sigma) and acquired using a FACSCanto II equipment and FACSDiva software (BD Biosciences). For intracellular detection of cytokines, leukocytes obtained from lungs were stimulated for 6 hours in complete RPMI medium containing 50 ng/mL phorbol 12-myristate 13-acetate, 500 ng/mL ionomycin (Sigma-Aldrich), and 3 mM monensin (eBioscience). Next, cells were labeled for surface molecules and then treated according to the manufacturer’s protocol for intracellular staining using the Cytofix/Cytoperm kit (BD Biosciences). Cells were washed twice with staining buffer, resuspended in 100 μl, and an equal volume of 2% formalin was added to fix the cells. A minimum of 50,000 events were acquired on FACScanto II flow cytometer (BD Biosciences) using the FACSDiva software (BD Biosciences). Lymphocytes were gated as judged from forward and side light scatter. For Treg cell characterization, FACS plots or histograms were gated on live CD4^+^FoxP3^GFP+^ cells. Otherwise, gated cells were measured for CD4 or CD8 expression and then for the respective surface or intracellular molecules.

### RNA isolation and cDNA synthesis

Lungs or sorted Treg cells were homogenized in TRIzol reagent using tissue grinders. Phase separation was achieved following addition of 0.2 ml chloroform per ml of TRIzol and centrifugation at 12000×*g* for 15 min at 4°C. The upper aqueous RNA phase was transferred to a fresh tube and further purified using Ultraclean Tissue & Cells RNA Isolation Kit (MO BIO Laboratories) according to the manufacturer’s protocol. RNA purity and concentration were assessed on a NanoDrop ND-1000 spectrophotometer. An amount of 1 μg total RNA was reverse transcribed in a 20 μl reaction mixture using the High Capacity RNA-to-cDNA kit (Applied Biosystems) following the manufacturer’s instructions.

### Real-time quantitative polymerase chain reaction (RT–PCR)

The cDNA was amplified using TaqMan Universal PCR Master Mix (Applied Biosystems) and predeveloped TaqMan assay primers and probes (IL-10, Mm00439614_m1; Tbet, Mm00450960_m1; GATA3, Mm00484683_m1; RORγC, Mm01261022_m1; Foxp3, Mm00475162_m1; PD-L1, Mm00452054_m1; CCR5, Mm01963251_s1; CCR6, Mm99999114_s1; Granzyme B, Mm00442834_m1; GAPDH, Mm 99999915_g1; all from Applied Biosystems). Probes were labelled with 6-carboxyfluorescein (FAM) at their 5′-terminal end. Data were normalized to GAPDH gene expression. *Taq*Man PCR assays were performed on a MxP3000P QPCR System and data were developed using the MxPro QPCR software (Stratagene).

### Cytokine detection

Lungs from mice infected with *P*. *brasiliensis* were aseptically removed and individually disrupted in 5 mL of PBS. Supernatants were separated from cell debris by centrifugation at 3000×*g* for 20 min and stored as 1ml-aliquots with protease inhibitors at -80°C. The levels of IL-2, IL-4, IL-6, IL-10, IL-12, IL-17, IL-23, IFN-γ and TGF-β were measured by capture enzyme-linked immunosorbent assay (ELISA) with antibodies pairs purchased from eBioscience. The ELISA procedure was carried out according to the manufacturer’s protocol. The concentrations of cytokines were determined with reference to a standard curve for a series of twofold dilutions of murine recombinant cytokines. Plates were read using a spectrophotometric plate reader (VersaMax, Molecular Devices). Protein concentration of lysates was determined using a bicinchoninic acid protein assay kit (Pierce).

### Statistics

Values represent means ± standard deviations (SD). Unless otherwise stated, group means were compared by Student’s *t* test. Differences between survivals were compared by log-rank test. All statistical analyses were performed using the software GraphPad Prism (GraphPad Software, Inc.).

## Results

### Conventional CD4^+^ T lymphocytes and Treg cells accumulate in the lungs and become activated during PCM

**Fig 1:**
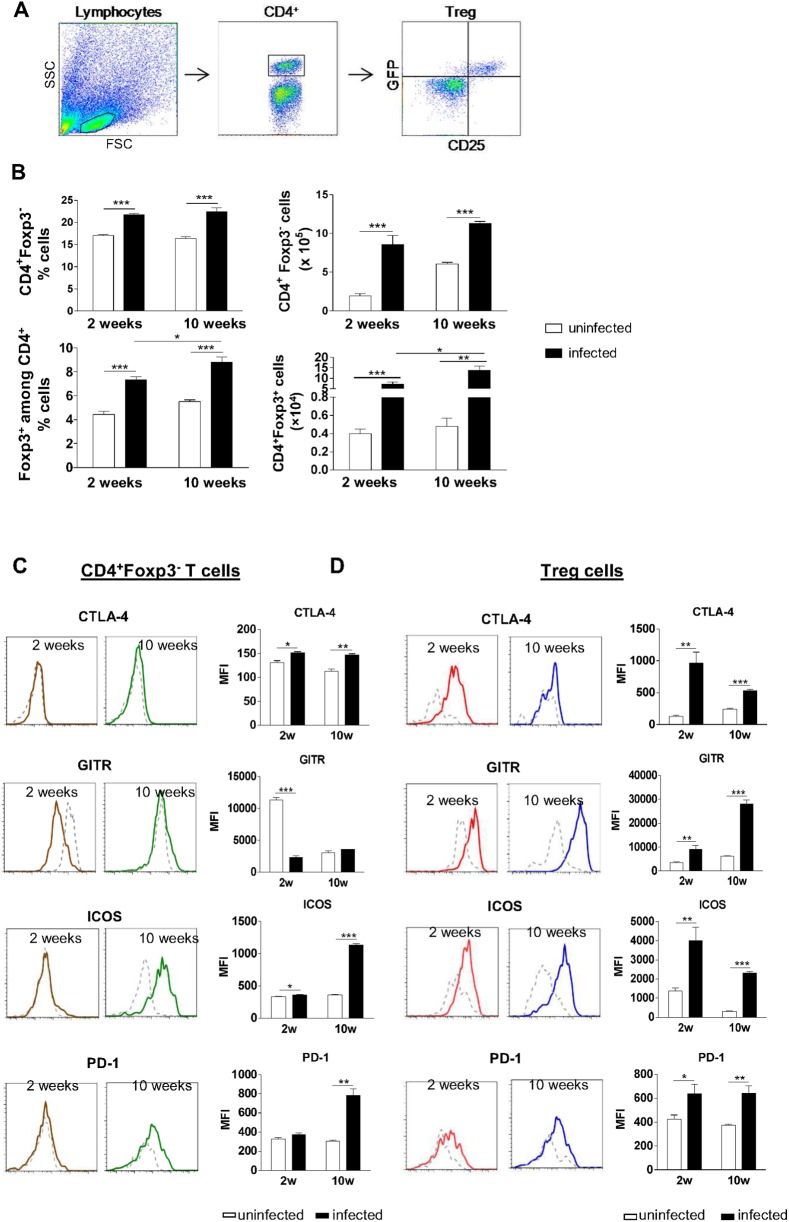
Characterization of conventional CD4^+^ T cells as well as Treg cells during pulmonary PCM. (A) Representative FACS plots demonstrating the gating strategy for CD4^+^ T cells and T reg cells. (B) Expansion of CD4^+^ T lymphocytes during infection with *P*. *brasiliensis*. Percent (left) and total number (right) of CD4^+^Foxp3^-^ T cells and CD4^+^Foxp3^+^ T cells were analyzed by flow cytometry in the lungs of infected and uninfected C57BL/6 Foxp3^GFP^ mice. Numbers indicate mean ± SD for at least five mice per group and are representative of three independent experiments (* *p* < 0.05; ** *p* < 0.005; *** *p* < 0.001). (C, D) Flow cytometric characterization of CD4^+^Foxp3^-^ T cells (C) and CD4^+^Foxp3^+^ T cells (D) indicate an activated status at the site of infection of both cell subsets, as shown by CTLA-4, GITR, ICOS, and PD-1 expression. Solid lines represent infected mice, whereas dashed lines indicate uninfected mice. Median fluorescence intensity (MFI) values were calculated from all CD4^+^Foxp3^-^ T cells or CD4^+^Foxp3^GFP+^ T cells. Bars reflect mean ± SD of three independent experiments with five mice per group (* *p* < 0.05; ** *p* < 0.005; *** *p* < 0.001).

To assess the expansion of conventional CD4^+^ T cells as well as Treg cells in both early and chronic phases of PCM, Foxp3^GFP^ mice were infected with 1 × 10^6^ Pb yeast cells. The percent and the absolute number of CD4^+^Foxp3^-^ T lymphocytes and of CD4^+^ T cells expressing Foxp3 in the lungs were determined by flow cytometry after 2 and 10 weeks of infection. Gated cells in lung homogenates are shown in Fig 1A. Infected mice showed significantly higher frequency and number of CD4^+^Foxp3^-^ and CD4^+^Foxp3^+^ T cells compared to uninfected control mice, in both periods analyzed (Fig 1B). Furthermore, both the percent and the total number of Foxp3^+^ cells were higher at week 10 compared with week 2 (Fig 1B).

In order to verify whether the expansion of CD4^+^Foxp3^-^ and CD4^+^Foxp3^+^ T cells was associated with a change in their activation status, both cell subsets were stained for surface molecules that are associated with an activated phenotype and analyzed by flow cytometry after 2 and 10 weeks of infection. Fig 1C shows that infection with Pb18 led to increased expression of most of the activation markers CTLA-4, GITR, ICOS and PD-1 on the surface of CD4^+^Foxp3^-^ and CD4^+^Foxp3^+^ T cells both in the early and in the late stages of infection.

### Treg cells in the lungs display a natural-like phenotype

**Fig 2:**
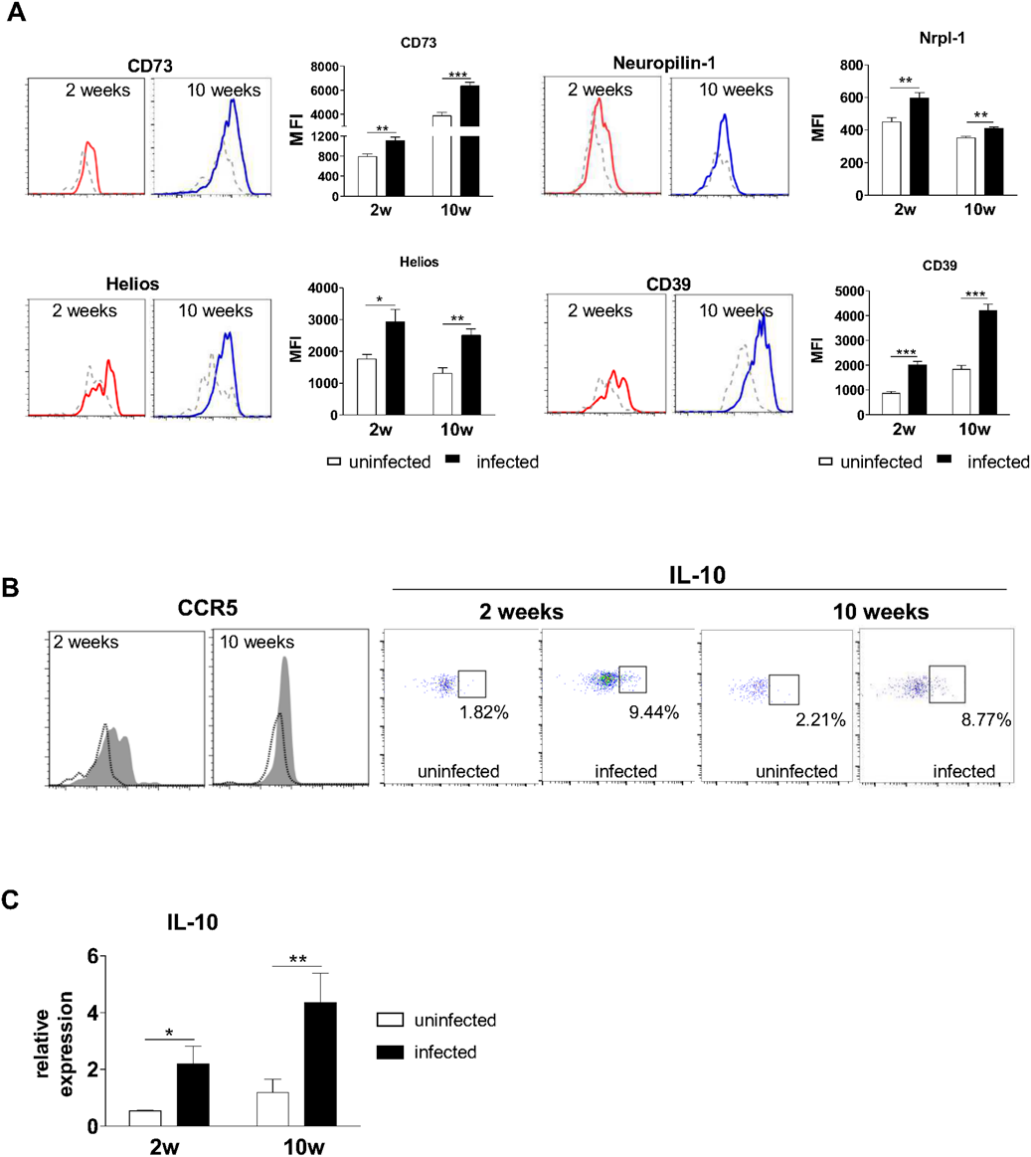
Treg cells display a natural-like phenotype and express typical surface and intracellular markers during infection. (A) Expression of surface and intracellular markers suggest a natural Treg-associated phenotype, as demonstrated by increased expression of Neuropilin-1, CD39, CD73 and Helios. Solid lines represent infected mice, whereas dashed lines indicate uninfected mice. (B) Treg cells in the lungs exhibit augmented expression of the chemokine receptor CCR5 and the suppressive cytokine IL-10 after infection with Pb18. Cells were stained for surface CCR5 or permeabilized and stained for intracellular IL-10. Histograms were gated on Foxp3^GFP+^ cells and show infected (shaded area) and uninfected (black line) mice. Dot plots display the frequency of Foxp3^GFP+^ cells expressing IL-10. Histograms and dot plots are representative of three independent experiments with at least five mice per group. (C) IL-10 mRNA levels in Treg cells increase after infection, as analyzed by RT-PCR. Treg cells from lungs of uninfected and infected mice (weeks 2 and 10 after infection) were isolated and total RNA was extracted. After cDNA synthesis, RT-PCR was performed using primers for IL-10. Bars represent mean ± SD from at least 5 mice per group. One representative experiment is shown (* *p* < 0.05; **p < 0.005).

After observing that Treg cells acquire an activated phenotype during PCM, we then wanted to investigate whether these cells detected in the site of infection had originated in the thymus (natural Tregs, nTregs) or in the periphery (induced Treg, iTreg). It has been recently demonstrated that nTregs display higher levels of surface Neuropilin-1, CD39, CD73 and the transcription factor Helios when compared to iTregs [reviewed by 17]. Lung Treg cells from infected animals showed higher expression of these molecules, as analyzed by flow cytometry, at weeks 2 and 10 post-infection, suggesting that at least some Treg cells present in the site of infection display an nTreg phenotype (Fig 2A). We then investigated CCR5 expression on Treg cell surface, since several lines of evidence demonstrate that Treg cells expressing CCR5 migrate to inflamed tissues through recognition of CCR5 ligands [8,18]. In fact, Treg cells from infected mice displayed higher expression of CCR5 compared with uninfected control mice at both early and late phases of infection (Fig 2B). To assess whether production of IL-10 by Treg cells changed during the course of infection, lung cells were stimulated *ex vivo* for 6 h with PMA and ionomycin in the presence of Brefeldin A for the last 4 h. As shown in Fig 2 B, a higher frequency of Treg cells in the lungs of infected animals was able to secrete IL-10 upon restimulation compared with cells from uninfected control mice (Fig 2B). This finding was further confirmed by assessing the mRNA levels of IL-10 in Treg cells (Fig 2C). Infected mice showed significantly increased IL-10 mRNA levels after 2 and 10 weeks of infection compared with Treg cells from uninfected mice.

### Treg cell depletion inhibits pathogen growth and dissemination

**Fig 3:**
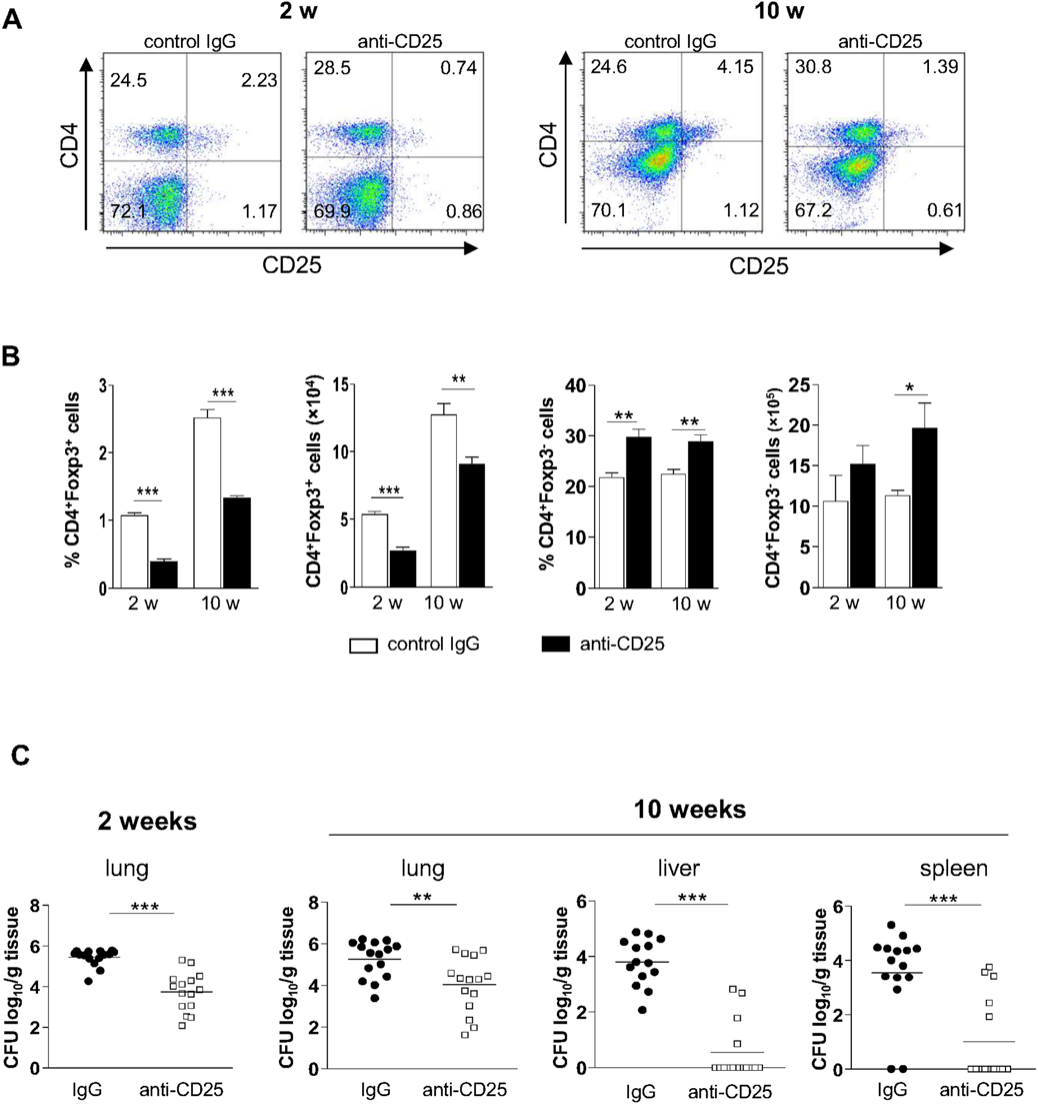
Ablation of Treg cell leads to decreased fungal loads and diminished dissemination. (A) Groups of five C57BL/6Foxp3^GFP^ mice were given anti-CD25 or control IgG antibodies and inoculated with 10^6^ *P*. *brasiliensis* yeast cells. The frequency of lung-derived CD4^+^CD25^+^ cells at weeks 2 and 10 post-infection is indicated. (B) Frequency and number of conventional CD4^+^ T cells as well as of Treg cells in the lungs at weeks 2 and 10 after infection following administration of control IgG or anti-CD25 antibody. Bars represent mean ± SD of three independent experiments with four to six mice per group (** *p* < 0.005; *** *p* < 0.001). (C) Fungal burden in organs of mice infected with Pb18 and treated with anti-CD25 or control IgG, after 2 (left) and 10 (right) weeks of infection. Horizontal bars indicate the mean value in each group (**p < 0.05, ***p < 0.005; n ≥ 15).

After characterizing the phenotype of Treg cells that migrate to the site of infection, we wanted to determine whether these cells would modulate the immune response to *P*. *brasiliensis*. Our previous studies using resistant (A/J) and susceptible (B10.A) mice have shown a protective effect of early Treg cell depletion by anti-CD25 antibodies (PC61). We then explored the effect of this treatment in C57BL/6 mice, which have an intermediate pattern of resistance to *P*. *brasiliensis* infection [19]. C57BL/6 Foxp3^GFP^ mice were treated with anti-CD25 or control rat IgG and infected with Pb18, according to a protocol previously established by our group [9]. As shown in Fig 3A, anti-CD25 treatment diminished the frequency of CD4^+^CD25^+^, but not of CD4^+^CD25^-^ T cells. At week 10 post infection, this effect was still observed. At week 2 post-infection, the frequency and the number of CD4^+^CD25^+^Foxp3^+^ T cells was significantly lower in groups of mice receiving anti-CD25 when compared with IgG-treated mice. This decrease in Treg cell frequency and number could still be observed by week 10, albeit at a lower extent (Fig 3B). In contrast, the frequency and number of CD4^+^Foxp3^-^ T cells increased upon anti-CD25 treatment (Fig 3B). After 2 weeks of infection, anti-CD25 treated animals showed significantly diminished fungal burdens in the lungs compared to mice receiving control IgG, as determined by CFU assays (Fig 3C). Even at week 10 post-infection, mice given anti-CD25 showed small numbers of CFU in the lungs, associated with impaired dissemination to other target organs, such as liver and spleen (Fig 3C).

### Treg-depleted mice show reduced lesion size

**Fig 4:**
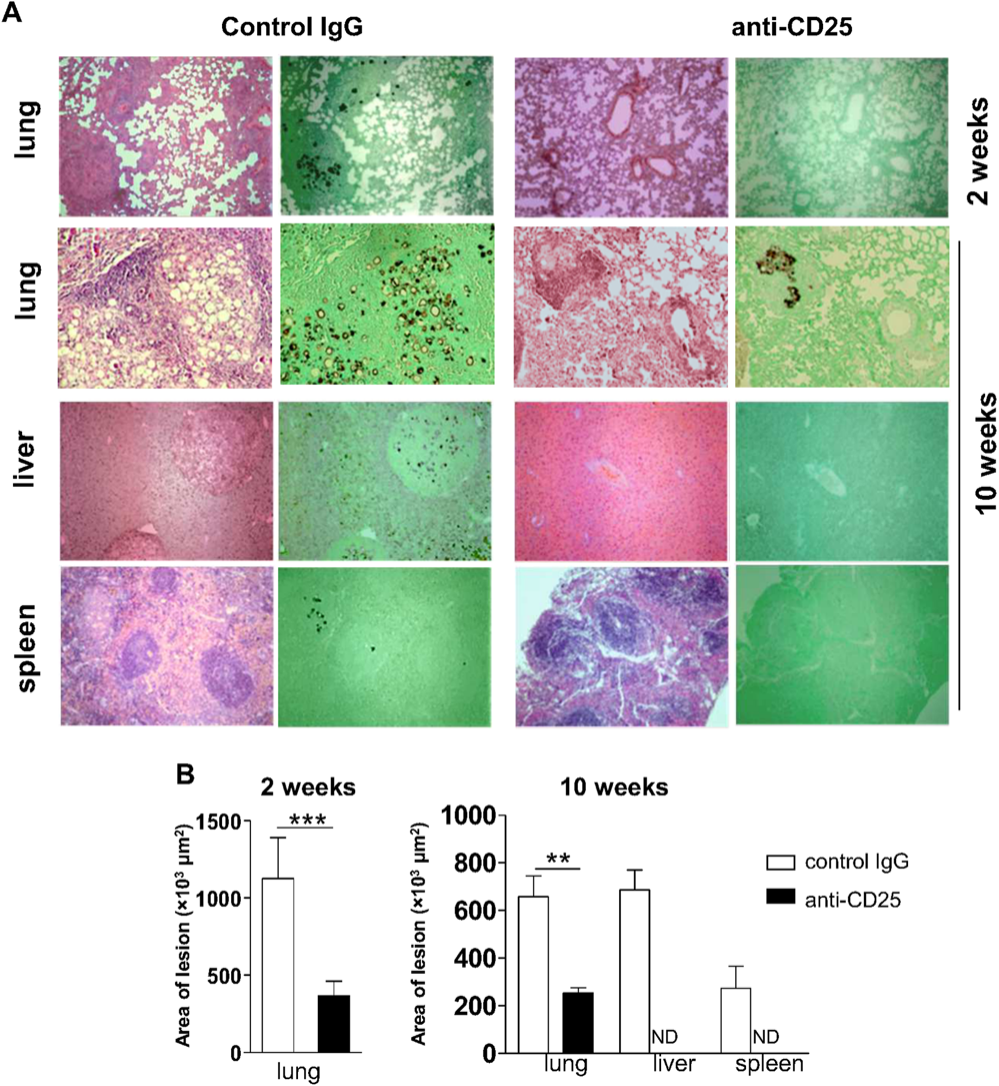
Histopathological analysis of target organs from infected mice treated with anti-CD25 or control IgG antibodies. (A) Organ micrographs after 2 (top) and 10 (bottom) weeks of infection with *P*. *brasiliensis*. Sections were stained with H&E or Grocott and examined with a magnification of 100×. (B) Morphometric analysis of organ lesions from mice treated with anti-CD25 or control IgG after 2 (left) and 10 (right) weeks of infection. Areas were quantified from photomicrographs of organ sections. Data are means ± SD of three independent experiments with similar results (n ≥ 6; ***p < 0.005; ND = not detectable).

Histological examination of organ sections performed after 2 and 10 weeks of infection showed that control mice exhibited more severe pathology, with great number of lesions, granulomas containing augmented numbers of yeast particles and some degree of tissue damage (Fig 4A). By contrast, mice receiving anti-CD25 antibody displayed well preserved lung parenchyma besides reduced amounts of lesions and yeast cells than mice treated with control IgG. Livers and spleens of anti-CD25-treated animals showed normal morphology after 10 weeks of infection, opposite to IgG-treated mice, which showed yeasts-containing granulomas in livers and spleens (Fig 4A). Morphometric analyses performed after 2 and 10 weeks of infection revealed larger lesion areas in the lungs of control mice by week 2, compared with anti-CD25-treated mice (Fig 4B). At week 10 post-infection, IgG-treated animals showed granulomas in the lungs, livers and spleens, contrasting to anti-CD25-trated mice, which exhibited smaller granulomas in the lungs and virtually no lesions in livers and spleens (Fig 4B).

### Depletion of Treg cells leads to increased Th1/Th2/Th17 responses

**Fig 5:**
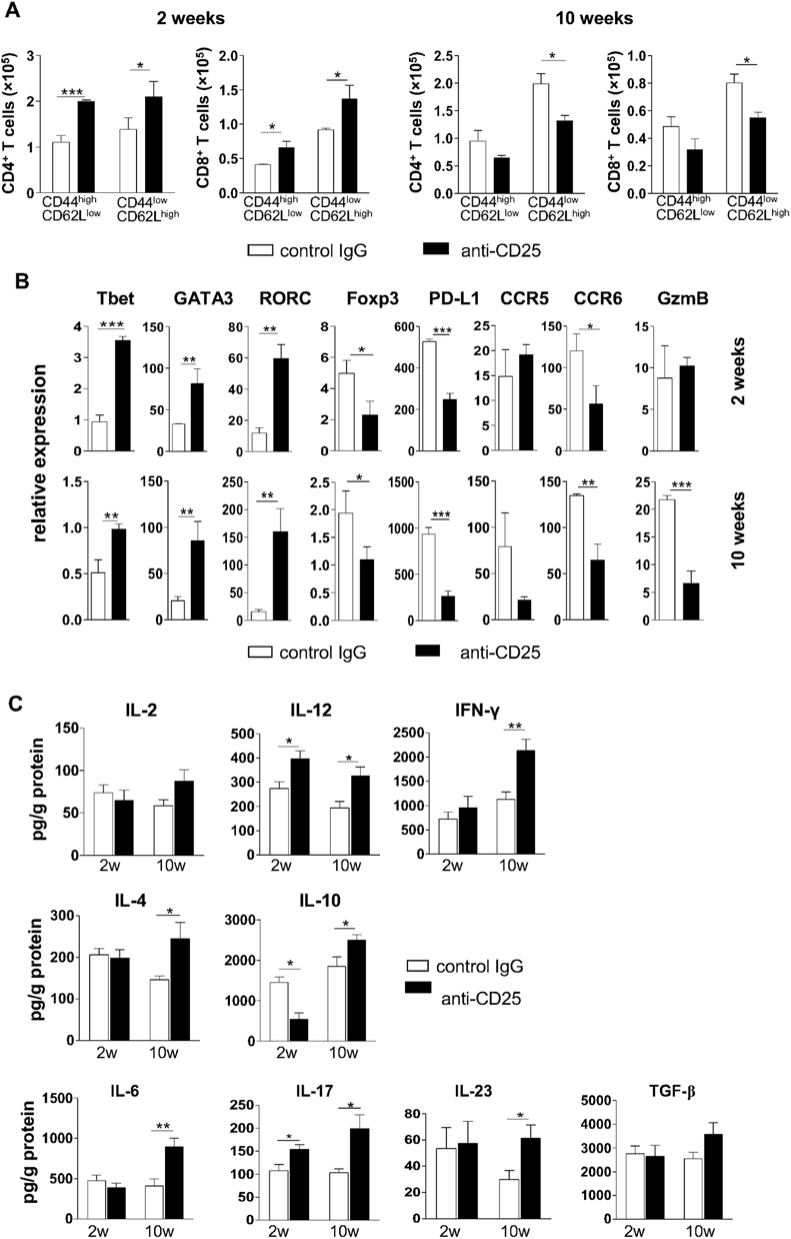
Depletion of Treg cells induces balanced Th1/Th2/Th17 immunity in both stages of infection. (A) Quantification of activated/memory and naïve CD4^+^ and CD8^+^ T lymphocytes in lungs from mice treated with anti-CD25 or control IgG and analyzed after 2 (left) and 10 (right) weeks of infection. (B) mRNA relative expression of Tbet, GATA3, RORγC, Foxp3, PD-L1, CCR5, CCR6 and Granzyme B in lung cells of mice treated with anti-CD25 or control IgG, after 2 (top) and 10 (bottom) weeks of infection. **(C)** Cytokine quantitation by ELISA in lung homogenates from mice treated with anti-CD25 or control IgG. Bars show mean ± SD from at least five mice per group and are representative of three independent experiments (*p< 0.05, **p < 0.005, ***p <0.001).

In order to verify whether depletion of Treg cells at the beginning of infection changed the influx of T cells to the lungs, the expression of surface markers indicative of activated or naïve T cells were examined by flow cytometry. At week 2 post-infection, a more intense influx of activated/memory (CD44^high^CD62L^low^) and naïve (CD44^low^CD62L^high^) CD4^+^ and CD8^+^ T lymphocytes was observed in the lungs of mice receiving anti-CD25 in comparison with IgG-treated mice (Fig 5A). After 10 weeks of infection, where Treg-depleted mice showed a significant reduction in fungal loads, the number of T cells in the lungs of Treg-depleted mice diminished in relation to control mice, but this difference was only significant with respect to naïve T cells (Fig 5A).

Next, we wanted to investigate whether treatment with anti-CD25 could modify the expression profile of genes associated with Treg and T cell subtypes in the lungs. Expression of total lung mRNA was analyzed after 2 and 10 weeks of infection with P. *brasiliensis* by RT-PCR. As shown in Fig 5B, we observed increased mRNA levels of the Th1/Th2/Th17-related major transcription factors Tbet, GATA3, and RORγC and diminished expression levels of Foxp3, CCR6 and PD-L1 after 2 weeks of infection. No significant changes in mRNA levels of CCR5 and Granzyme B were detected in this early phase of infection. Later on in infection, at week 10, the increase in the mRNA levels of Tbet, GATA3 and RORγC paralleled the reduction in expression levels of the Treg-associated molecules Foxp3, PD-L1, CCR5, CCR6 and Granzyme B (Fig 5B).

The presence of Th1/Th2/Th17-associated cytokines was evaluated in lung homogenates of anti-CD25- and control IgG-treated mice in both early and late phases of infection. After 2 weeks of infection, an increase in the levels of IL-12 and IL-17 and a reduction in the levels of IL-10 were observed in Treg-depleted mice, as compared to control mice (Fig 5C). By week 10, anti-CD25-treated animals showed significant increased levels of IL-12, IFN-γ, IL-4, IL-10, IL-6, IL-17 and IL-23 when compared with IgG-treated mice (Fig 5C). Altogether, our data suggest that the temporary depletion of Treg cells at the beginning of infection increased the expansion of Th1/Th17 cells and, at a later stage, augmented Th1/Th2/Th17 mixed-type immune responses.

### Adoptive transfer of T cell subsets demonstrates that Treg cells collaborate for the protective effects of CD4^+^Foxp3^-^ T cells, but fail to promote pathogen eradication

**Fig 6:**
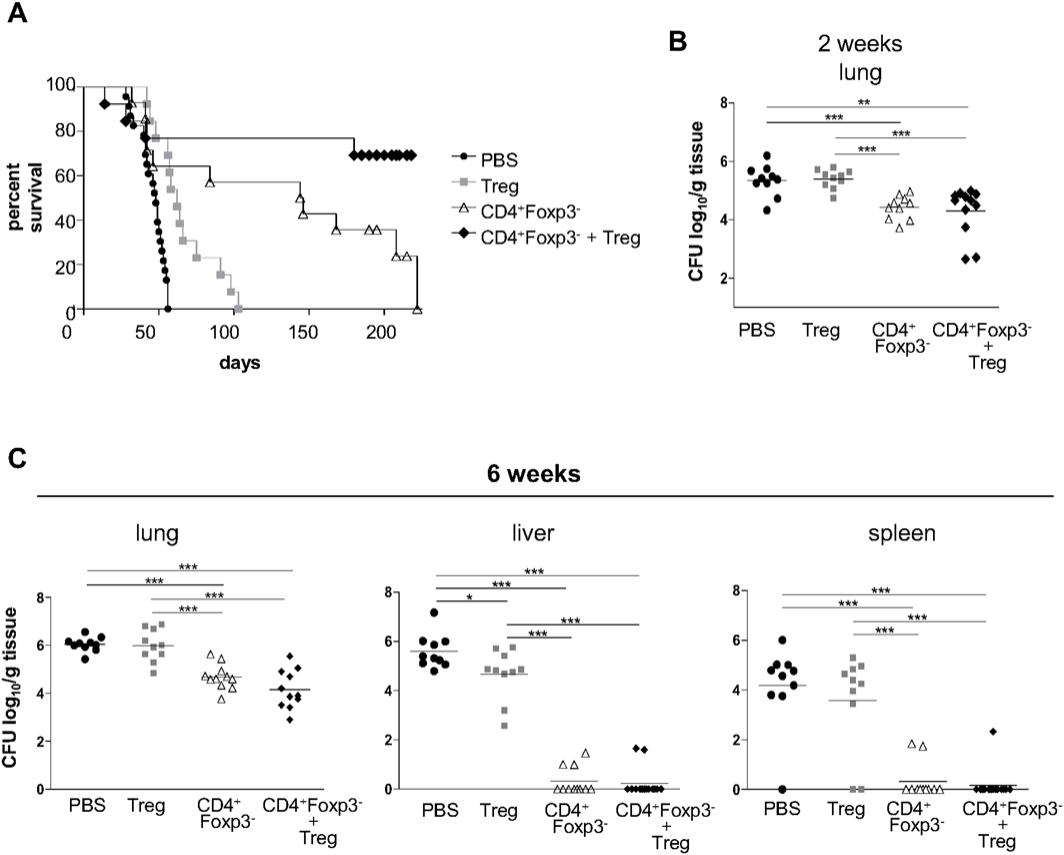
Adoptive transfer of Treg cells improves the protective effect of CD4^+^Foxp3^-^ T cells. (A) Survival curves of Rag1^-/-^ mice infected with Pb18 after adoptive transfer of 2×10^5^ Treg cells, 2×10^6^ CD4^+^Foxp3^-^ T cells, 2×10^5^ Treg cells + 2×10^6^ CD4^+^Foxp3^-^ T cells, or vehicle only (*p* < 0.05 between PBS and Treg; *p* < 0.001 between PBS and CD4^+^Foxp3^-^ T cells; *p* <0.001 between PBS and CD4^+^Foxp3^-^ + Treg; *p* < 0.05 between Treg and CD4^+^Foxp3^-^; *p* < 0.001 between Treg and CD4^+^Foxp3^-^ + Treg; n ≥ 13). (B and C) Analysis of disease severity through recovery of CFU from lungs, livers and spleens from Rag1^-/-^ mice infected with Pb18 after adoptive transfer of 2×10^5^ Treg cells, 2×10^6^ CD4^+^Foxp3^-^ T cells, 2×10^5^ Treg cells + 2×10^6^ CD4^+^Foxp3^-^ T cells, or vehicle only. Horizontal bars indicate the mean value in each group (* = *p* < 0.05; ** = *p* < 0.005; *** = *p* < 0.001; n ≥ 10).

Once the effects of Treg cell depletion had been determined on the course of PCM using C57BL/6 Foxp3^GFP^ mice, the disease outcome of mice reconstituted with diverse T cell subpopulations was then evaluated. To do so, CD4^+^Foxp3^-^ and CD4^+^Foxp3^+^ T lymphocytes from C57BL/6 Foxp3^GFP^ mice were isolated and adoptively transferred to B and T cell deficient Rag1^-/-^ mice, which were subsequently infected with Pb18. Survival of *P*. *brasiliensis*-infected Rag1^-/-^ mice was registered daily over a 220-day period (Fig 6A). Animals receiving only vehicle before infection showed the largest mortality rates, with a mean survival time of 45.7 days. Mice given Treg cells exhibited a statistically significant greater survival time than those injected with vehicle, and had a mean survival period of 66.5 days. Mice reconstituted with CD4^+^ Foxp3^-^ T cells prior to infection showed a mean survival of 126.8 days. Interestingly, mice receiving CD4^+^ Foxp3^-^ T cells + Treg cells displayed the highest survival time. At the end of the observation period, most animals were still alive, so that the mean survival of this group remained undetermined.

Fungal growth and dissemination in Rag1^-/-^ mice were evaluated after 2 and 6 weeks of infection (Fig 6B and 6C). By week 2, fungal burden in the lungs of mice reconstituted with Treg cells was similar to those receiving vehicle only, and significantly greater than in mice given CD4^+^Foxp3^-^ T cells and CD4^+^Foxp3^-^ T cells + Tregs. With respect to these last two groups, there was no statistical difference in fungal burden, revealing that in the early phase of infection, the presence of CD4^+^ T cells *per se* can lead to a better fungal clearance (Fig 6B). At the late phase of infection, mice receiving CD4^+^Foxp3^-^ T cells or CD4^+^Foxp3^-^ T cells + Treg cells showed significantly lower fungal burdens in the lungs when compared to mice given Treg cells or vehicle, besides very low dissemination to liver and spleen (Fig 6C). In some mice, fungal burden was kept under the detection limit of the assay, suggesting sterile eradication. Interestingly, Treg-reconstituted mice showed lower CFU counts in livers compared to mice receiving vehicle.

### Adoptive transfer of Treg cells limits excessive inflammation caused by CD4^+^Foxp3^-^ T cells

**Fig 7:**
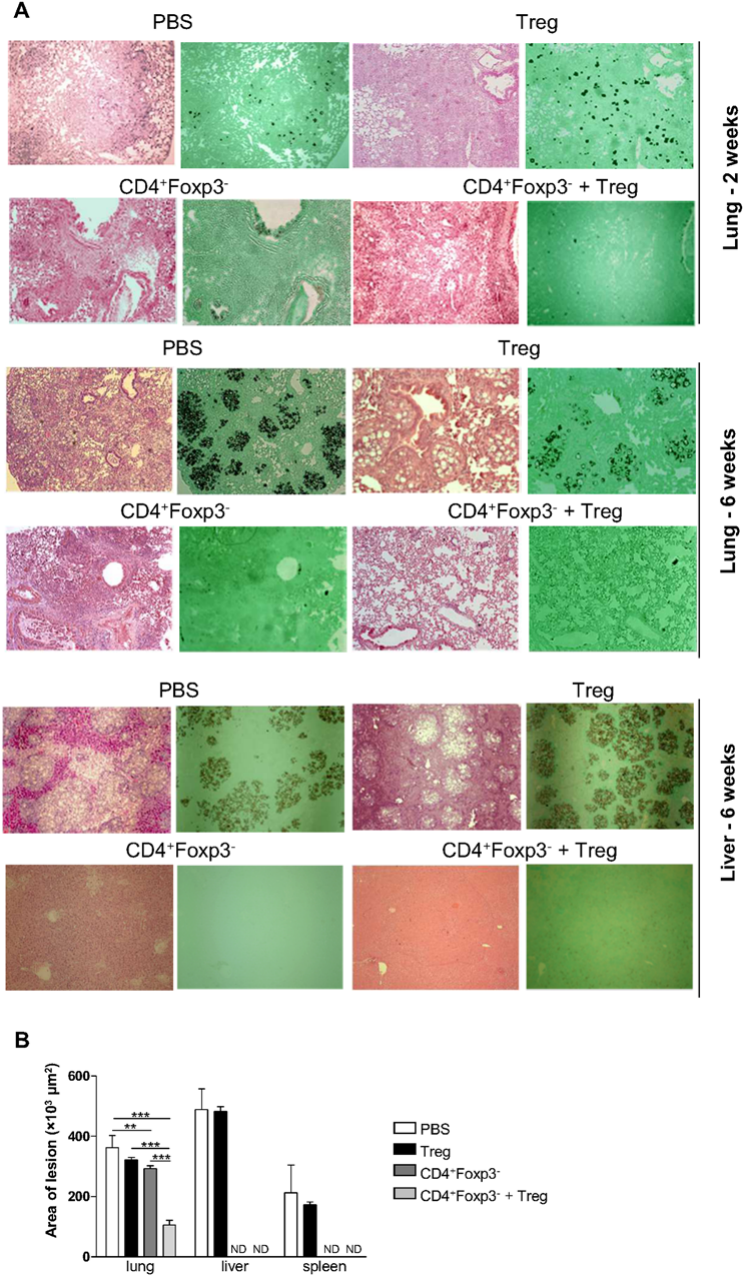
In the absence of Tregs, mice show exacerbated inflammatory responses. (A) Photomicrographs of lungs, livers and spleens from Rag1^-/-^ mice infected with Pb18 after adoptive cell transfer of Treg, CD4^+^Foxp3^-^ T cells and Treg + CD4^+^Foxp3^-^ T cells. Organ sections were stained with H&E and Grocott and examined under a light microscope with a magnification of 100×. Morphometric analysis of lesions in lungs, livers and spleens from Rag1^-/-^ mice after 6 weeks of infection with Pb18. (B) Areas of lesions were quantified from photomicrographs of organ sections. Data represent the mean ± SD from three experiments (n ≥ 5; *p < 0.05; ** *p* < 0.005; ***p < 0.001; ND = not detectable).

Disease severity was also evaluated in organ sections from all groups of mice after 2 and 6 weeks of infection. At week 2 post-infection, lungs of mice receiving either vehicle or Treg cells showed yeasts-containing granulomas and substantial destruction of the lung architecture (Fig 7A). By contrast, mice reconstituted with CD4^+^Foxp3^-^ T cells or CD4^+^Foxp3^-^ T cells + Treg cells exhibited diffuse inflammation in the lung tissue, containing disperse or no yeast cells. After 6 weeks of infection, lungs of mice receiving vehicle or Treg showed a vast number of granulomas containing a high number of fungal particles compromising a large area of pulmonary tissue (Fig 7A). Lungs of mice reconstituted with CD4^+^Foxp3^-^ T cells still showed intense and disseminated cellular infiltration in the alveolar spaces and in the peribronchiolar and perivascular regions, but with few detectable yeast cells. Lungs of mice given CD4^+^Foxp3^-^ T cells + Treg cells showed signs of resolved inflammation and low number of fungal particles. Livers from mice receiving vehicle or Treg cells showed a great number of granulomas containing yeast cells, whereas livers from mice reconstituted with CD4^+^Foxp3^-^ T cells or CD4^+^Foxp3^-^ T cells + Treg cells displayed preserved tissue architecture (Fig 7A).

The areas of lesions were measured based on the organ sections corresponding to week 6 post-infection (Fig 7B). Organs of mice given vehicle or Treg cells showed lesions with similar size. Mice receiving CD4^+^Foxp3^-^ T cells showed large lesion areas in the lungs and no detectable lesions in livers and spleens, whilst mice reconstituted with CD4^+^Foxp3^-^ T cells + Treg displayed significantly smaller lesions in the lungs when compared with all other groups.

### Treg cells induce enhanced Th1/Th2/Th17 immunity without increased T cell infiltration

**Fig 8:**
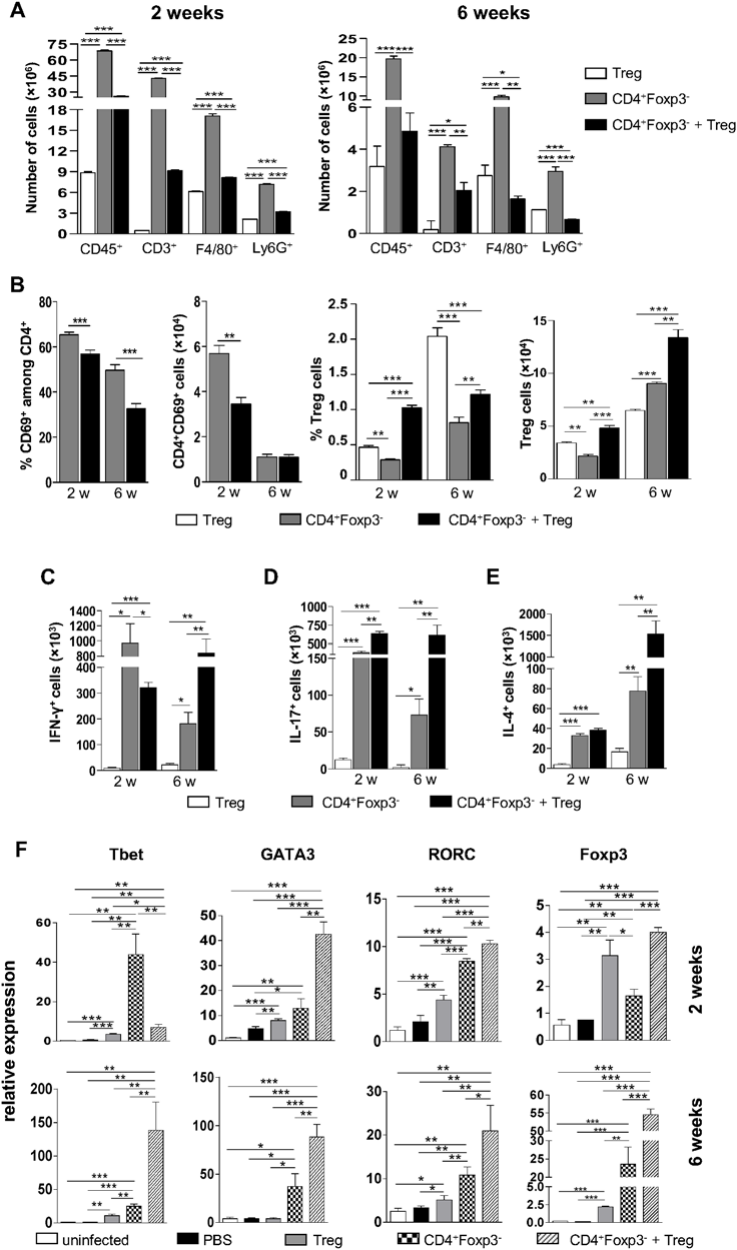
Treg cells limit inflammation and increase the differentiation of Th1, Th2 and Th17 cells. Rag1^-/-^ mice were reconstituted with different T cell subpopulations, infected with *P*. *brasiliensis* and sacrificed at the time points indicated. Lungs were excised and single cells were stained as described in Materials and Methods section. (A) Number of total leukocytes, lymphocytes, macrophages and granulocytes in the lungs of infected Rag1^-/-^ mice given Treg cells, CD4^+^Foxp3^-^ T cells or CD4^+^Foxp3^-^ T cells + Treg cells. (B) Frequency and number of CD4^+^CD69^+^Foxp3^-^ T lymphocytes and Treg cells in the lungs of infected Rag1^-/-^ mice previously reconstituted with Treg cells, CD4^+^Foxp3^-^ T cells or CD4^+^Foxp3^-^ T cells + Treg cells. (C-E) Numbers of CD4^+^ T lymphocytes producing IFN-γ, IL-17 and IL-4 in reconstituted and infected Rag1^-/-^ mice. (F) Quantitative PCR analysis of major transcription factors of T cells in the lungs of Rag1^-/-^ mice, as determined by RT-PCR after 2 and 6 weeks post-infection. Rag1^-/-^ mice were reconstituted with the different T cell subpopulations or vehicle only, infected with *P*. *brasiliensis* or left uninfected, and sacrificed after 2 and 6 weeks. Lungs were removed, RNA was extracted, reverse transcribed and the amplified cDNA was used for RT-PCR, as described previously. All bars indicate mean ± SD from at least 5 mice per group and are representative of three independent experiments (* *p* < 0.05; **p < 0.005; ***p < 0.001).

In order to examine the inflammatory reaction caused by *P*. *brasiliensis* infection, leukocyte recruitment to the lungs of Rag1^-/-^ mice receiving the different T cell subpopulations was assessed. Mice receiving CD4^+^Foxp3^-^ T cells showed significantly higher numbers of lymphocytes, macrophages and granulocytes in the lungs at weeks 2 and 6 post-infection as compared to mice receiving Treg cells alone or in combination with CD4^+^Foxp3^-^ T cells (Fig 8A). At week 2, mice reconstituted with CD4^+^Foxp3^-^ T cells + Treg cells exhibited increased numbers of inflammatory cells in the lungs than mice receiving isolated Treg cells. However, at week 6, the group given Treg cells displayed a more intense recruitment of macrophages and granulocytes to the site of infection than the group receiving the combination of CD4^+^Foxp3^-^ T cells and Treg cells (Fig 8A). These data are in agreement with the histopathological findings shown in Fig 7A.

To further investigate the mechanisms involved in protection against disease, T lymphocytes from reconstituted Rag1^-/-^ mice were analyzed by flow cytometry after 2 and 6 weeks of infection. As shown in Fig 8B, mice receiving CD4^+^Foxp3^-^ T cells exhibited a greater number of infiltrating activated (CD69^+^) CD4^+^ T cells in the lungs compared to mice given CD4^+^Foxp3^-^ T cells + Treg cells. By week 6, both mice groups displayed equivalent numbers of activated CD4^+^ T cells, which were lower than those observed at week 2. The subpopulation CD4^+^CD69^+^ could not be detected in lungs of Rag1^-/-^ mice receiving Treg cells only. With respect to the Treg cell population, mice receiving CD4^+^Foxp3^-^ T cells + Treg cells showed the highest number of Tregs at both periods analyzed. At week 6, the number of Tregs was higher in mice given CD4^+^Foxp3^-^ T cells than in mice transferred with Treg cells only, as opposed to week 2, although the frequency of these cells was still greater in the group receiving CD4^+^Foxp3^-^ T cells.

Intracellular staining of CD4^+^ T lymphocytes revealed that mice reconstituted with CD4^+^Foxp3^-^ T cells and CD4^+^Foxp3^-^ T cells + Treg showed augmented production of IFN-γ, IL-17 and IL-4, at weeks 2 and 6 post-infection, when compared with mice receiving Treg cells only (Fig 8C–8E). At week 2, mice reconstituted with CD4^+^Foxp3^-^ T cells exhibited the highest numbers of cells expressing IFN-γ (Fig 8C), while mice given CD4^+^Foxp3^-^ T cells + Treg displayed larger numbers of cells producing IL-17 (Fig 8D). At week 6 post-infection, mice receiving CD4^+^Foxp3^-^ T cells + Treg showed the greatest numbers of cells producing IFN-γ, IL-17 and IL-4 (Fig 8C–8E).

In order to determine the mRNA levels of the major T cell transcription factors (Foxp3, Tbet, GATA3 and RORγC) after reconstitution of Rag1^-/-^ mice, RT-PCR analyses of total lung RNA were performed after 2 and 6 weeks of infection (Fig 8F). By week 2, the expression of Tbet was notably augmented in the group receiving CD4^+^Foxp3^-^ T cells, followed by the group given CD4^+^Foxp3^-^ T cells + Treg cells and then by the group reconstituted with Treg cells only. At week 6, Tbet mRNA levels were higher in lungs of mice receiving CD4^+^Foxp3^-^ T cells + Treg. Levels of GATA3 mRNA were augmented in mice given CD4^+^Foxp3^-^ T cells + Treg cells, followed by mice receiving CD4^+^Foxp3^-^ T cells and then by mice injected with Treg cells only. At week 6, GATA3 mRNA was still higher in mice reconstituted with CD4^+^Foxp3^-^ T cells + Treg, followed by the group receiving CD4^+^Foxp3^-^ T cells only. RORγC mRNA levels at week 2 and 6 post-infection were higher in mice reconstituted with CD4^+^Foxp3^-^ T cells + Treg cells, followed by mice given CD4^+^Foxp3^-^ T cells and then by mice receiving Treg cells. With respect to Foxp3, mice reconstituted with the combination of the two cell subpopulations displayed the highest levels of this transcription factor, followed by mice receiving Treg and then by mice given CD4^+^Foxp3^-^ T cells at week 2. By week 6, Foxp3 mRNA levels in lungs of mice receiving CD4^+^Foxp3^-^ T cells were higher than in mice reconstituted with Treg only, and still lower than in mice given CD4^+^Foxp3^-^ T cells + Treg cells (Fig 8F).

## Discussion

Using loss- and gain-of-function experimental approaches for the manipulation of Treg cells *in vivo*, we could demonstrate the role of these cells in the inhibition of both the advantageous and deleterious aspects of the immune response subsequent to infection with *P*. *brasiliensis*. In this work, we used C57BL/6 Foxp3^GFP^ transgenic mice to track the development, localization and function of Treg cells at early and late stages of infection. Subsequently, the effects of depletion of Treg cells with anti-CD25 antibody at the early stage of *P*. *brasiliensis* infection were investigated. Further, the course of this disease was accompanied after adoptive transfer of CD4^+^ Foxp3^-^, CD4^+^Foxp3^+^ T cells, or a combination of both subpopulations, to immunodeficient Rag1^-/-^ mice.

Using Foxp3^GFP^ knock-in mice, we observed a significant accumulation of Treg cells in the lungs of infected mice, at the early and late periods of infection. At least in part, this accumulation was due to migration of nTreg cells to the site of infection, as evidenced by enhanced expression of Neuropilin-1, CD39, CD73, Helios and CCR5. Although it has been postulated that Neuropilin-1 is an excellent marker to characterize nTreg cells in diverse immune settings [20] and CD39/CD73 have been extensively used as Treg cell markers [reviewed by 21], as far as we know, no specific marker for nTregs in a context of infectious disease has been described. However, the set of markers used in our study to analyze Treg cells strongly suggest that these cells display a nTreg phenotype. The increased frequency of Foxp3^+^ cells observed in the lungs of mice was similar to the acute infection with respiratory sincitial virus (RSV) [22], but differs from data obtained for *M*. *tuberculosis* infection, in which the number of Treg cells increased significantly in the sites of infection, but their percentage among CD4^+^ T cells did not alter significantly [23]. Besides accumulating in the site of infection, Treg cells showed an altered phenotype, becoming activated in the course of disease. These findings were also observed in conventional CD4^+^ T cells.

The signals that induce Treg cell activation during infection with *P*. *brasiliensis* are not yet fully elucidated. This is particularly intriguing with respect to nTreg cells, since they are supposed to recognize autoantigens. Nevertheless, Fulton et al., observing this phenomenon in an experimental model of infection by RSV, have speculated that nTregs could recognize tissue-specific antigens in the context of inflammation caused by infection, or that they could be unspecifically activated by recognition receptors, such as Toll-like receptors (TLRs) and cytokine receptors [22]. This could also be true in our infection model, since it has been demonstrated that cell wall components of *P*. *brasiliensis* are recognized by pattern recognition receptors such as TLR2, TLR4 and dectin-1 [10–12,24,25]. Upon infection, the increase in the expression of surface markers such as GITR, ICOS and PD-1, which have been associated to the suppressive activity of Tregs, is consistent with findings from others, such as in the infection with RSV or *M*. *tuberculosis* [22,23]. Furthermore, secretion of IL-10 by Treg cells at the site of infection may suggest the existence of suppressive mechanisms influencing the ability of effector T cells to promote pathogen eradication. Our group has recently shown the deleterious effects of IL-10 on the host´s ability to cure *P*. *brasiliensis* infection [26].

The accumulation of Tregs in the lungs prompted us to investigate whether these cells would have the potential to limit the ability of the immune system to effectively respond to infection. Thus, Treg cells were depleted with anti-CD25 antibody (clone PC61). This treatment does not completely eliminate Treg cells, since there are Foxp3^+^ cells which show low or no expression of CD25 [27]. This may be the case of a cell population that has recently bound IL-2, since the IL-2 receptor protein complex (of which CD25 takes part) is internalized after binding of IL-2 and further recycled to the cell surface [28]. Yet the drastic effects observed after treatment with anti-CD25 indicate that partial elimination of Treg cells is sufficient to disturb the balance between regulatory and effector cells [29]. It has been demonstrated that elimination of Treg cells after PC61 administration occurs through phagocytosis of this cell population upon recognition of the rat IgG1 by FCRγIII on the surface of phagocytes [29]. After treatment with anti-CD25, we observed a reduction in the number and frequency of Treg cells, which was consistent with the reduced fungal burden and increased influx of memory/effector and naïve CD4^+^ and CD8^+^ T cells to the lungs. By week 10 of infection, anti-CD25-treated mice showed diminished numbers of CD4^+^ and CD8^+^ T lymphocytes in the lungs, indicating that the reduction in the number of Tregs did not cause exacerbated inflammatory reactions. In addition, the reduced fungal loads appear to have contributed to this finding. The protective effect of Treg cells depletion was also demonstrated by the dramatic decrease of fungal burdens in the dissemination organs. The decrease of the Foxp3^+^ population, still observed at week 10, caused remarkable effects on the dissemination of the fungus to other target organs.

In this work, cytokine determination showed that anti-CD25-treated mice produced lower amounts of IL-10 in the lungs when compared to control animals after 2 weeks of infection. Since IL-10^-/-^ mice infected with Pb18 have been shown to control disease more efficiently than WT mice [26], the lower IL-10 amounts associated to the depletion of the Treg cell population has possibly contributed to the augmented resistance to infection observed in the animals treated with anti-CD25. At the late phase of infection, the increase in the levels of cytokines typical for Th1 Th2 and Th17 responses indicates lack of polarization of the immune response and was consistent with the increase in the mRNA levels of the transcription factors Tbet, GATA3 and RORγC. The reduction in the levels of Treg-associated molecules, such as Foxp3, CCR5, CCR6, PD-L1 and Granzyme B, maintained even after week 10 post-infection, corroborates the beneficial effects of the early anti-CD25 treatment.

The effects of Treg cell activity on the course of different infectious diseases depend on the pathogen involved. For instance, Treg depletion in mice infected with *Toxoplasma gondii* led to greater susceptibility and higher parasite burden [30,31]. In the RSV infection model, Treg ablation elicited more severe infection, more intense weight loss and greater morbidity [22]. Mice infected with *Pneumocystis* showed augmented lung damage and exacerbated Th2-type responses after treatment with anti-CD25 [32]. In other experimental models, like infection by Influenza A virus, treatment with anti-CD25 did not alter the course of infection, since no changes in mortality, weight loss and viral clearance were observed [33]. By contrast, in an experimental model of infection by *M*. *tuberculosis*, Treg cell depletion led to decreased bacterial burden and increased numbers of activated T cells in the lungs [23]. Similar effects were observed in mice infected with *Salmonella enterica* after depletion of Treg cells early in infection [34]. Our findings with C57BL/6 Foxp3^GFP^ mice are in line with previous data from our group showing that susceptible B10.A and resistant A/J mice treated with anti-CD25 exhibited lower fungal burdens in the target organs, less severe pathology and higher production of Th1/Th2/Th17 cytokines [9].

Recent studies using diverse approaches of murine PCM brought indirect evidences for both the deleterious and protective effects of Treg cells. For instance, the deleterious role of Tregs have been previously demonstrated using CD28^-/-^ and WT mice. CD28-suficient mice showed persistent presence of Treg cells, leading to deactivation of inflammatory cells, production of anti-inflammatory cytokines and higher mortality rates compared with CD28^-/-^ mice [35]. Further, reduced numbers of Treg cells were shown to diminish disease severity in both resistant A/J and susceptible B10.A mice, as demonstrated by treatment with anti-CD25 antibodies [9]. These data are supported by a study using CCR5^-/-^ mice, which exhibited decreased frequency of Tregs in granulomatous lesions and consequently more limited fungal growth and dissemination in relation to their WT counterparts [8]. Nevertheless, diminished numbers of Treg cells have been associated with uncontrolled inflammatory responses and increased tissue pathology in TLR2^-/-^ mice, suggesting a protective role of Tregs [11]. A similar disease outcome was observed in another study conducted by our group, in which TLR4-sufficient mice displayed impaired expansion of Treg cells, augmented influx of activated T lymphocytes and macrophages to the lungs and consequent more severe fungal infection than TLR4-deficient mice [12].

Using a more direct approach to analyze the function of Treg cells, adoptive transfer experiments to Rag1^-/-^ mice were performed. The observation that Treg cell transfer before infection with *P*. *brasiliensis* led to higher survival rates in relation to mice receiving vehicle indicates a certain, albeit low, degree of protection, possibly by limiting excessive tissue pathology caused by pathogen-mediated inflammation. This fact might also explain the greater survival of mice given CD4^+^Foxp3^-^ T cells + Treg cells in comparison to mice receiving CD4^+^Foxp3^-^ T cells only. With respect to CFU counts, we can assume that CD4^+^ T cells *per se* are sufficient to limit fungal growth and prevent dissemination to liver and spleen, and that Tregs do not negatively interfere in this process. Interestingly, these results are in contrast to our previous data on Treg cell ablation after anti-CD25 treatment of B10.A, A/J and Foxp3^GFP^ mice, in which lower numbers of Tregs led to diminished fungal loads and dissemination [9, this work]. This finding also contrasts to the reports of Kursar et al, who demonstrated that, during infection by *M*. *tuberculosis*, mice reconstituted with CD4^+^CD25^-^ + CD4^+^CD25^+^ T cells showed higher pathogen burden than mice receiving CD4^+^CD25^-^ T cells [36]. However, in a murine model of infection by *C*. *albicans*, mice given both cell subpopulations displayed lower fungal burden compared to those receiving CD4^+^CD25^-^ T cells only [37]. The slightly lower number of CFU found in the liver of Treg-reconstituted mice in comparison to vehicle-given mice might suggest the conversion of part of this population into Foxp3^+^RORγC^+^ T cells, a newly identified cell population which expresses both Treg- and Th17-associated molecules and is thought to exert both regulatory and effector mechanisms [reviewed by 38]. In fact, cytokine and mRNA analyses revealed both IL-17 and RORγC expression in mice given Treg cells.

Cytokine production analysis by flow cytometry revealed, at week 2 post-infection, higher amounts of IFN-γ in lungs of mice receiving CD4^+^Foxp3^-^ T cells in comparison to the other groups, which is consistent with the elevated levels of Tbet expression found in the lungs of mice from this group. These findings are in agreement with observations from Kursar et al. (2007) in an infection model with *M*. *tuberculosis* [36]. Thus, in the absence of Treg cells, there is a pronounced Th1 polarization of CD4^+^ T cells. By week 6, mice given CD4^+^Foxp3^-^ T cells + Treg cells exhibited higher production of IFN-γ, IL-4 and IL-17 when compared to the other groups, which correlates with the higher mRNA levels of Tbet, GATA3 and RORγC. This balanced expression of the different Th cell subpopulations at the late phase of infection suggest the occurrence of a more efficient immune response without increased influx of T cells and might explain the higher survival rates observed in this group. The observed enhanced inflammation in the lungs of Rag1^-/-^ mice reconstituted with CD4^+^Foxp3^-^ T cells was analogous to the findings from a previous study performed by our group in which IL-12 administration to B10.A mice led to diminished *P*. *brasiliensis* dissemination to liver and spleen, but induced increased lung inflammation [39]. In that work, mice given IL-12 showed a significant augment in IFN-γ production at the beginning of infection and reduction of Th1/Th2-related cytokines at the late stage of infection, similarly to what was observed in the present study for Rag1^-/-^ mice injected with CD4^+^Foxp3^-^ T cells. In the latter group, the presence of IFN-γ early in infection led to a higher control of fungal burden and dissemination, but induced a greater influx of lymphocytes, macrophages and granulocytes, causing a strong inflammation that could no longer be properly controlled or limited, even though a cell subpopulation expressing Foxp3 had been detected. These results are corroborated by a study in which Rag2^-/-^ mice were infected with *Pneumocystis carinii* after receiving CD4^+^CD25^+^ T cells, CD4^+^CD25^-^ T cells, or a combination of both subpopulations. Mice reconstituted with CD4^+^CD25^-^ T cells were able to eliminate the pathogen, but died of acute pneumonia consequent of intense infiltration of inflammatory cells. This scenario was not observed in mice receiving CD4^+^CD25^+^ T cells, either isolated or in combination with CD4^+^CD25^-^ T cells, since Tregs limited exacerbated inflammation [4]. Our data on histological examination of lung sections also support the hypothesis of pathology resulting from excessive inflammation elicited by CD4^+^Foxp3^-^ cells which were in turn triggered by Pb infection.

This is the first work that provides direct evidences for the deleterious and protective effects of Treg cells in pulmonary PCM through its suppressive effect on the immune response and control of excessive inflammation. This study also sheds light on the function of Treg in human PCM, since, to our knowledge, all published works have associated the presence of these cells to severe disease outcomes. In this way, the elucidation of the role of Tregs in PCM is crucial to establish new therapeutic approaches for treatment of PCM patients.
